# Psychosocial Factors Associated with Healthy and Unhealthy Interpregnancy Intervals

**DOI:** 10.1089/heq.2017.0017

**Published:** 2018-03-01

**Authors:** Ruth Young, Wendy G. Lane, Stacey B. Stephens, Bronwyn W. Mayden, Renee E. Fox

**Affiliations:** ^1^ Department of Psychiatry, University of Maryland School of Medicine, Baltimore, Maryland.; ^2^ Department of Epidemiology and Public Health, University of Maryland School of Medicine, Baltimore, Maryland.; ^3^ Department of Pediatrics, University of Maryland School of Medicine, Baltimore, Maryland.; ^4^ University of Maryland School of Social Work, Baltimore, Maryland.; ^5^ Division of Quality & Health Outcomes, Center for Medicare and Medicaid Services, Baltimore, Maryland.

**Keywords:** intimate partner violence, pregnancy, psychosocial, risk factors, social support, substance abuse

## Abstract

**Purpose:** To examine the influence of psychosocial factors, including anxiety, depression, social support, maternal substance abuse, and intimate partner violence (IPV) on interpregnancy intervals (IPIs).

**Methods:** B'more for Healthy Babies–Upton/Druid Heights is part of a citywide initiative to improve the health of at-risk pregnant women and their children. Participants with at least one prior birth completed baseline, postpartum, and 3-month follow-up surveys with questions about pregnancy, medical, and psychosocial history. Associations between IPI and the independent variables were assessed using chi-square analysis and analysis of variance. Multivariable multinomial logistic regression models examined significant associations while controlling for other independent variables and potential confounders.

**Results:** Participants with current IPV were more likely to have a short IPI (odds ratio [OR]=13.1; 95% confidence interval [CI]=1.07–158.9; *p*=0.04) than healthy IPI. Women with family social support were more likely to have a healthy IPI (OR=5.88, 95% CI=1.02–31.25, *p*=0.05) than those without family social support. Maternal anxiety and depression did not significantly influence IPI.

**Conclusion:** IPV increased the likelihood of having an unhealthy IPI among this population and family social support increased the likelihood of having a healthy IPI. Additional efforts to address IPV and enhance family social support may lead to improved pregnancy outcomes.

## Introduction

Short and long interpregnancy intervals (IPIs), less than 18 months and greater than 59 months, have been shown to significantly increase the risk of adverse maternal and neonatal outcomes, including low birth weight (LBW), preterm delivery, maternal anemia, and preeclampsia.^1–8^ Several hypotheses, including maternal nutrient and folate depletion, physiological depletion, and postpartum stress have been proposed as potential mediators in this relationship between unhealthy IPIs and adverse outcomes.^4–6,8^

Demographic characteristics associated with short IPIs include being of ethnic minority, young maternal age, unmarried status, low education level, history of reproductive loss, low socioeconomic status, and late initiation of prenatal care.^1,3,4,6,8–11^ Additionally, these demographic and several psychosocial risk factors (e.g., depression and anxiety, job strain, parenting stress, and intimate partner violence [IPV]) increase the risk for LBW and preterm birth.^12–15^

While multiple studies have looked at the relationship between psychosocial factors and adverse birth outcomes, few have examined how psychosocial factors such as mental health, IPV, substance abuse, or social support, might contribute to IPI length, which may serve as a potential target to prevent adverse birth outcomes.^1,6,16,17^ Determining any existing associations of these risk factors and IPI could provide better insight into how to improve IPI and subsequently prevent adverse pregnancy outcomes. This knowledge would be especially helpful in groups of vulnerable women, like those in low-income, low-education, predominantly minority inner city neighborhoods where these psychosocial factors and poor pregnancy outcomes are particularly pervasive.^18^

This study seeks to explore this area by examining whether anxiety, depression, lack of social support (family and friend), maternal substance abuse, and IPV contribute to unhealthy IPIs among women enrolled in the Upton/Druid Heights B'more for Healthy Babies Program (BHB-U/DH).

## Methods

### BHB program

B'more for Healthy Babies is a Baltimore-based citywide initiative intended to improve the health of pregnant women and their newborns through media messages and community outreach. The citywide program supports two place-based initiatives, one of which is located in the Upton/Druid Heights neighborhood of West Baltimore. BHB-U/DH is coordinated and evaluated by a team of faculty and staff from an urban academic medical center in collaboration with a neighborhood Community Action Team.

BHB-U/DH staff conducts extensive community outreach to identify pregnant Upton/Druid Heights residents and encourage them to enroll in the BHB-U/DH program. Enrolled women must be pregnant at the time of enrollment and live within the borders of the Upton/Druid Heights neighborhood. They receive an assessment of medical, psychosocial, and other needs, partner with a community health worker to help address their needs, and participate in an evidence-based prenatal care (PNC) curriculum.

### Data collection

BHB-U/DH tracks maternal and child outcomes for research and clinical purposes through data collected at program entry (during pregnancy), postpartum, and every 3 months until the child reaches 18 months of age. The questionnaires were completed during these periods by participants with the assistance of community health workers and cover topics, including pregnancy, medical, and psychosocial aspects of the participants' lives. It is from these forms that data were ascertained for this study. All variables were measured by self-report and all participants provided written, signed informed consent.

### Participant overview

The 76 women enrolled in BHB-U/DH from April 2011 through April 2014 who had at least one prior birth were eligible for inclusion. The baseline information collected at the time of enrollment (ranging from 1 to 9 months gestation) included history of prior births, current pregnancy complications, previous and current health problems, current living situation, and other psychosocial factors that might affect pregnancy outcome. The psychosocial risk factors examined included IPV, substance abuse, depression, anxiety, and social support from family and friends. Birth history information included number of previous births, prior preterm births, LBW babies, and fetal loss. History of current pregnancy information included pregnancy intent, plan to breast feed, and month of initiation of PNC.

### Determination of dependent variable: IPI

We used a standard definition of IPI as the interval between the delivery date of the preceding live birth and the conception date of the index pregnancy and divided the participants into three categories according to Jose-Agudelo's meta-analysis: short (<18 months), healthy (>18 months and <59 months), and long (>59 months).^2,19^ We broadly refer to IPIs outside of the 18–59 month range as “unhealthy IPIs”.

Because there were no direct IPI questions in the forms, we calculated the value from information available from baseline and postpartum data forms. Available data included date of birth, whether the child was born early and, if so, the number of weeks early from postpartum forms and months since prior birth from the baseline form. Weeks of gestation were determined through a combination of participant estimation using last menstrual period and ultrasound. We calculated an approximate date of conception from the date of birth and number of weeks early. We then calculated the gestational age of each pregnancy at the time of baseline data collection. Lastly, we determined IPI by subtracting the number of months of gestation at baseline data collection from the months since prior birth.

For those participants who did not fill out postpartum data forms but did fill out the 3-month follow-up forms, we assumed a full-term pregnancy and estimated the date of conception by subtracting the sum of the age of the infant and 40 weeks. As before, we used this estimated conception date to determine IPI.

Nineteen participants completed baseline forms, but did not complete the postpartum or 3-month follow-up forms. All but one participant had identified the number of months since prior birth that fell clearly within a specific IPI category; that is, the interval was at least 9 months greater than the short IPI cutoff of 18 or 9 months less than the 59-month-long IPI cutoff. The final participant's entry for months since prior birth was 60 months, so she was placed in the healthy IPI category as we assumed that this individual was at least 4 weeks pregnant at the time of baseline data intake.

### Independent variables assessment: psychosocial factors

IPV, maternal substance abuse, social support, anxiety, and depression were evaluated by participant report through questions on the baseline data collection forms. We assessed current IPV using the screening question: “Have you ever been hurt or physically threatened by your current partner?”.^20^ We assessed social support using three screening questions focused on family social support, friend social support, and help with their baby: “Is there someone in your family you could talk to if you had a problem?”, “Is there someone outside your family you could talk to if you had a problem?”, and “Do you have someone who will help you care for your baby?”.

We defined substance abuse as one or more positive responses on the CAGE-AID screen.^21^ We evaluated anxiety by the medical history questionnaire, including those who self-identified as having a history of medically diagnosed anxiety. We identified depression using a dichotomous variable that incorporated history of and/or current clinical diagnosis in addition to screening questions: “Lately, have you often felt down, depressed, or hopeless?” and “Lately, have you felt very little interest or pleasure in things you used to enjoy?”.^20^ An affirmative response to any of these questions counted as positive for depression.

### Controls and confounders

We considered a number of variables that might confound the association between the predicted psychosocial risk factors and IPI, including race, healthcare coverage, maternal education, maternal smoking, housing security, maternal age, primary language spoken, and developmental delay. While the other control variables that we evaluated were either dichotomous or continuous, we separated education into four categories: less than a HS Diploma, HS Diploma only, HS Diploma and some college/technical school, and college/technical school graduate. We examined whether there were any statistically significant associations between these potential confounders and independent variables (current IPV, substance abuse, social support, anxiety, and depression), and between potential confounders and IPI.

### Data analysis

Participants were characterized by age, education, insurance coverage, race, primary language spoken, birth history, current pregnancy history, and housing security. We calculated mean and standard deviations (SDs) for continuous variables (age and previous births), and proportions for categorical and ordinal variables (all others).

Unadjusted associations between our categorical independent variables and IPI were examined using chi-square analyses and simple multinomial logistic regression. To examine the significance of associations between continuous variables and IPI, we used analysis of variance (ANOVA). Subsequently, we examined associations between potential confounders and IPI, and between potential confounders and independent variables using chi-square analyses for categorical variables and t-tests and ANOVA for continuous variables.

We used multivariable multinomial logistic regression to examine the relationship between our independent variables and IPI (short vs. healthy IPI vs. long), controlling for potential confounders. Potential confounders were included in the multivariable models if the associations between the potential confounder and the independent variables were significant at *p*<0.10.

Because there was little or no variation among participants, we did not include race (nearly all African American), healthcare coverage (nearly all insured by Medicaid), and neighborhood variables in the analysis. We controlled for maternal age in all analyses and created separate multinomial regression models for each of our independent variables. We included other independent variables as potential confounders in the models when the *p*-value for the association with the independent and dependent variable was <0.10.

Microsoft Excel was used to perform univariate and bivariate analyses and STATA was used for simple and multivariable multinomial regression.

The University of Maryland Baltimore Human Research Protections Office reviewed and approved the intervention and evaluation.

## Results

### Participant demographics

At the time of this study (April 2011–April 2014), 111 women had participated in the program and 76 were eligible for this analysis. Ninety-seven percent of our participants were African American and 94.7% were native English speakers. Nearly all were insured by Medicaid. The average age (SD) of participants was 26.7 (5.3) years and 85.3% of participants had stable housing. Thirty-six percent had not graduated from high school, 17.3% had a high school diploma only, and 46.7% had some additional postsecondary education (technical school or college).

Participants had an average of 2.2 prior births (SD=1.6), with 29.3% having at least one previous preterm birth, 20% having at least one previous LBW baby, and 33.3% having at least one prior fetal loss. For their current pregnancy, 81.1% were unintentional and 81.3% initiated PNC in the first trimester, whereas 8% had yet to start PNC or started in the third trimester (Table 1).

**Table 1.** **Demographic Characteristics of Participants (*n*=76)**

Demographic category	Short IPI n *(%) or mean (SD)*	Intermediate IPI n *(%) or mean (SD)*	Long IPI n *(%) or mean (SD)*	Total n *(%) or mean (SD)*	*p*
Mean age (*n*=76)	24.3 (5.2)	25.2 (4.6)	30.4 (4.6)	26.71 (5.3)	<0.001
Education: (*n*=75)					0.38
<HS diploma	5 (31.3%)	13 (37.1%)	9 (37.5%)	27 (36%)	
HS diploma only	6 (37.5%)	4 (11.4%)	3 (12.5%)	13 (17.3%)	
HS diploma + some	4 (25%)	11 (31.4%)	8 (33.3%)	23 (30.7%)	
college/tech school					
College/technical grad	1 (6.3%)	7 (20%)	4 (16.7%)	12 (16%)	
Insurance (*n*=75)					0.15
No insurance	1 (6.3%)	0 (0%)	0 (0%)	1 (1.3%)	
Have insurance	15 (93.8%)	36 (100%)	23 (100%)	74 (98.7%)	
Med. Assist	15 (100%)	36 (100%)	23 (100%)	73 (98.6%)	
Other	0 (0%)	0 (0%)	0 (0%)	1 (1.4%)	
Race (*n*=76)					0.37
African American	15 (93.8%)	36 (100%)	23 (95.8%)	74 (97.4%)	
White	1 (6.3%)	0 (0%)	1 (4.2%)	2 (2.6%)	
Language (*n*=76)					0.22
English speaker	14 (87.5%)	34 (94.4%)	24 (100%)	72 (94.7%)	
Other language	2 (12.5%)	2 (5.6%)	0 (0%)	4 (5.3%)	
Birth history					
No. of prior births (*n*=76)	1.88 (1.21)	2.19 (1.56)	2.38 (2.04)	2.18 (1.64)	0.22
No. of prior preterm births (*n*=75)					0.25
None	10 (62.5%)	28 (80%)	15 (62.5%)	53 (70.7%)	
One or more	6 (37.5%)	7 (20%)	9 (37.5%)	22 (29.3%)	
No. of prior low birth weight babies: (*n*=75)					0.37
None	13 (81.3%)	30 (85.7%)	17 (70.8%)	60 (80%)	
One or more	3 (18.8%)	5 (14.3%)	7 (29.2%)	15 (20%)	
No. of prior fetal losses: (*n*=75)					0.98
None	11 (68.8%)	23 (65.7%)	16 (66.7%)	50 (66.7%)	
One or more	5 (31.3%)	12 (34.3%)	8 (33.3%)	25 (33.3%)	
Pregnancy					
Pregnancy intent (*n*=74)					0.75
Unintentional	14 (87.5%)	28 (80%)	18 (78.3%)	60 (81.1%)	
Intentional	2 (12.5%)	7 (20%)	5 (21.7%)	14 (18.9%)	
Breast feed plan (*n*=73)					0.84
Will not breast feed	5 (33.3%)	9 (25.7%)	7 (30.4%)	20 (27.4%)	
Plan to breast feed	10 (66.7%)	26 (74.3%)	16 (39.6%)	53 (69.7%)	
Start of PNC (*n*=75)					0.76
First trimester	13 (81.3%)	27 (77.1%)	21 (87.5%)	61 (81.3%)	
Second trimester	1 (6.3%)	5 (14.3%)	2 (8.33%)	8 (10.7%)	
Third trimester/not started yet	2 (12.5%)	3 (8.6%)	1 (4.2%)	6 (8%)	
Housing security (*n*=75)					0.29
Does not have home	4 (25%)	3 (8.6%)	4 (16.7%)	11 (14.7%)	
Has a home	12 (75%)	32 (91.4%)	20 (83.3%)	64 (85.3%)	

IPI, interpregnancy interval; SD, standard deviation; HS, high school; PNC, prenatal care.

### Bivariate analysis

In examining demographic characteristics for associations with IPI by chi-square tests or ANOVA, age was the only demographic characteristic found to be significantly associated with IPI (*p*<0.001) (Table 1).

Upon chi-square analysis of associations between the independent variables (IPV, substance abuse, social support–family, social support–friend, help with baby, depression, and anxiety) and IPI, we found that substance abuse (*p*=0.03) and family social support (*p*=0.02) were significantly associated with IPI and that there was not a significant association between IPV and IPI (*p*=0.09) (Table 2).

**Table 2.** **Chi-Square Analysis of Psychosocial Factors Versus Interpregnancy Interval**

	Short IPI *n* (%)	Healthy IPI *n* (%)	Long IPI *n* (%)	Total *n* (%)	*p*
Current IPV					0.09
No	10 (62.5)	31 (86.1)	21 (87.5)	62 (81.6)	
Yes	6 (37.5)	5 (13.9)	3 (12.5)	14 (18.4)	
Substance abuse					0.03
No	10 (66.7)	34 (94.4)	18 (75)	62 (82.7)	
Yes	5 (33.3)	2 (5.6)	6 (25)	13 (17.3)	
Family social support					0.02
No	10 (62.5)	8 (22.9)	8 (33.3)	26 (34.7)	
Yes	6 (37.5)	27 (77.1)	16 (66.7)	49 (65.3)	
Friend social support					0.83
No	4 (25)	10 (27.8)	5 (20.8)	19 (25)	
Yes	12 (75)	26 (72.2)	19 (79.2)	57 (75)	
Help with baby					0.18
No	5 (31.3)	8 (22.9)	2 (8.3)	15 (20)	
Yes	11 (68.8)	27 (77.1)	22 (91.7)	60 (80)	
Depression					0.70
No	4 (26.7)	14 (38.9)	9 (37.5)	27 (36)	
Yes	11(73.3)	22 (61.1)	15 (62.5)	48 (64)	
Anxiety					0.18
No	7 (46.7)	26 (72.2)	17 (70.8)	50 (66.7)	
Yes	8 (53.3)	10 (27.8)	7 (29.2)	25 (33.3)	

IPV, intimate partner violence.

We performed simple multinomial logistic regression analyses for these three associations with IPI (Table 3). Participants with substance abuse had a more than eight times increased risk of having a short IPI (odds ratio [OR]=8.5, 95% confidence interval [CI]=1.43–50.7; *p*=0.02) and an almost six times higher risk of having a long IPI (OR=5.67; 95% CI=1.04–31.0; *p*=0.05) versus a healthy IPI. Women who reported having family social support were less likely to have a short IPI than a healthy IPI (OR=0.17; 95% CI=0.05–0.64; *p*=0.008).

**Table 3.** **Simple Multinomial Logistic Regression for Significant Psychosocial Factors**

	OR (short vs. healthy)	95% CI	*p*	OR (long vs. healthy)	95% CI	*p*
Current IPV	3.72	0.93–14.9	0.06	0.89	0.19–4.11	0.88
Substance abuse	8.5	1.43–50.7	0.02	5.67	1.04–31.0	0.05
Family social support	0.17	0.05–0.64	0.008	0.59	0.19–1.89	0.38

OR, odds ratio; CI, confidence interval.

When we included confounding factors in the multivariable multinomial logistic regression (Table 4), we found that those with IPV were 13 times more likely to have a short IPI than a healthy IPI (OR=13.07; 95% CI=1.07–158.94; *p*=0.04). Those with substance abuse were more likely to have a short IPI (OR=10.7; 95% CI=0.78–146.28; *p*=0.08) or long IPI (OR=12.37; 95% CI=0.94–162.93; *p*=0.06) than a healthy IPI. These comparisons were not statistically significant. Lastly, those with family social support were less likely to have a short IPI than a healthy IPI (OR=0.18; 95% CI=0.032–0.99; *p*=0.05).

**Table 4.** **Multivariate Multinomial Logistic Regression for Significant Psychosocial Factors**

	OR (short vs. healthy)	95% CI	*p*	OR (long vs. healthy)	95% CI	*p*
Current IPV^a^	13.07	1.07–158.94	0.04	1.42	0.10–21.20	0.80
Substance abuse^b^	10.7	0.78–146.28	0.08	12.37	0.94–162.9	0.06
Family social support^c^	0.18	0.032–0.99	0.05	0.71	0.15–3.39	0.58

^a^ Controlling for: family social support, substance abuse, age, anxiety, housing security, smoking, and education.

^b^ Controlling for: current IPV, family social support, smoking, age, education, and housing security.

^c^ Controlling for: substance abuse, current IPV, age, anxiety, smoking, and education.

## Discussion

Given that IPIs, shorter and longer than the healthy range (18–59 months), are significantly associated with adverse pregnancy outcomes, this study sought to examine what types of psychosocial factors, if any, might increase or decrease one's likelihood of having a healthy IPI.^2^

Our findings incompletely supported our hypotheses that maternal substance abuse, IPV, lack of social support, anxiety, and depression would increase one's likelihood of having an unhealthy IPI. Anxiety, depression, substance abuse, and some types of social support (friend social support and help with baby) were not significantly associated with short or long IPIs versus healthy IPIs. However, IPV was associated with an increased likelihood of having a short IPI and the presence of family social support was associated with a decreased likelihood of having a short IPI vs. a healthy IPI.

Our findings have been supported by several other studies. One Sub-Saharan study looking at personal history of IPV as the independent variable and “interbirth interval” as the primary outcome found that women with a history of IPV had an increased risk of short IPI compared with those women without an IPV history.^22^

The association between IPV and unintended pregnancy has also been demonstrated.^23^ These findings might be attributed to the partner's control over sexual activity and use of birth control. Multiple studies have shown that women experiencing IPV are less likely to obtain adequate PNC.^24,25^ Others have found an independent association between IPV and delivery complications and adverse maternal outcomes.^17,25^ More investigations are needed to further classify these associations between IPV and short IPIs and IPV and adverse maternal outcomes.

At least one other study has shown increased risk of rapid repeat pregnancies in women with inadequate family support.^26^ There is strong evidence that inadequate social support is an important risk factor for both antenatal and postpartum depression.^27–30^ While our study did not find a significant association between depression and IPI, these associations between inadequate family support and short IPIs and between inadequate family support and depression indicate the need for further examination of these factors.

There were several limitations to our study. Because of our relatively small sample size (*n*=76) our power was limited and some estimates were imprecise, with large CIs. Additionally, there were too few participants with certain risk factors, such as homelessness and lack of insurance, to be able to adequately assess any relationship with IPI. However, despite the small sample size, we were able to demonstrate that current IPV and lack of family social support were significantly associated with short IPI with a CI of 95%. Future studies might integrate results from other community programs under the same citywide initiative to increase the number of participants.

While IPI had to be estimated for some women and therefore could have been misclassified, very few of the women with missing information had an IPI range that straddled IPI categories. Integration of questions specifically about IPI into the questionnaire would increase accuracy.

Misclassification bias is another potential limitation as none of our screens was 100% sensitive or specific. Women with risk factors, such as depression and substance abuse, may have been missed by our screening questions, and some women without these problems may have screened positive. Our definition of anxiety, for example, only included women with a clinical diagnosis and thus may have been underidentified. This would be a bias to our results toward finding no difference in IPI in relation to anxiety problems. Additionally, stigma about substance abuse, IPV, or depression may have led to underreporting of these problems, again biasing results toward the null hypothesis. Despite these limitations in reporting, the fact that data were gathered by program community health workers in the context of a supportive, helping relationship likely raised the accuracy of reporting.

## Health Equity Implications

While other studies have demonstrated the association between psychosocial risk factors and adverse birth outcomes, this study was unique in its approach of looking at IPI as an end-point in itself given its role as a potential target to reduce these adverse outcomes and in looking at a range of potential psychosocial risk factors that might contribute to an unhealthy IPI. Our data lend additional weight to the importance of identifying and addressing psychosocial risks among women of childbearing age, particularly among those residing in high poverty communities. Focused interventions such as improved screening, tailoring of interventions during a PNC visit, and expansion of resources like BHB-U/DH, could potentially prevent short IPIs and improve birth outcomes.

The importance of family social support is evident from our findings. Unfortunately, given that social support from friends did not have a significant effect on IPI, it may be challenging to find alternate sources of support for women whose family members are unable to fill this need. However, our hope is that support from community outreach workers and other pregnant and parenting women will provide the necessary resources to achieve healthy IPIs in subsequent pregnancies. Ongoing analysis of B'more for Healthy Babies outcomes will be important in determining the effectiveness of nonfamilial social support.

While additional examination of the role of IPV and inadequate family social support in their relation to negative birth outcomes is needed to better understand the causal versus correlative relationships among IPI, psychosocial risk factors and negative birth outcomes, our study was successful in its aim to identify relevant psychosocial factors that might contribute to unhealthy IPIs as a potential target to improve pregnancy outcomes. Given our findings that IPV increased the risk of having an unhealthy IPI and family social support increased the likelihood of having a healthy IPI, efforts by programs like BHB/U/DH to address IPV and to enhance social support may lead to improved pregnancy outcomes among these vulnerable populations.

## Abbreviations Used

BHB-U/DH: Upton/Druid Heights B'more for Healthy Babies Program
CI: confidence interval
IPIs: interpregnancy intervals
IPV: intimate partner violence
LBW: low birth weight
OR: odds ratio
PNC: prenatal care
SD: standard deviations
